# Cytogenetic and Molecular Effects of Kaolin’s Foliar Application in Grapevine (*Vitis vinifera* L.) under Summer’s Stressful Growing Conditions

**DOI:** 10.3390/genes15060747

**Published:** 2024-06-06

**Authors:** Ana Carvalho, Lia-Tânia Dinis, Ana Luzio, Sara Bernardo, José Moutinho-Pereira, José Lima-Brito

**Affiliations:** 1 Plant Cytogenomics Laboratory, Department of Genetics and Biotechnology, Laboratorial Complex, University of Trás-os-Montes and Alto Douro (UTAD), Quinta de Prados, 5000-801 Vila Real, Portugal; jbrito@utad.pt; 2 Centre for the Research and Technology of Agro-Environmental and Biological Sciences (CITAB), University of Trás-os-Montes and Alto Douro (UTAD), Quinta de Prados, 5000-801 Vila Real, Portugal; liatdinis@utad.pt (L.-T.D.); aluzio@utad.pt (A.L.); sbernardo@utad.pt (S.B.); moutinho@utad.pt (J.M.-P.); 3 Institute for Innovation, Capacity Building and Sustainability of Agri-Food Production (Inov4Agro), University of Trás-os-Montes and Alto Douro (UTAD), Quinta de Prados, 5000-801 Vila Real, Portugal

**Keywords:** aluminum silicate, antioxidant response, cell cycle regulation, leaf mitosis, quantitative real-time PCR (qPCR)

## Abstract

Grapevine varieties from “Douro Superior” (NE Portugal) experience high temperatures, solar radiation, and water deficit during the summer. This summer’s stressful growing conditions induce nucleic acids, lipids, and protein oxidation, which cause cellular, physiological, molecular, and biochemical changes. Cell cycle anomalies, mitosis delay, or cell death may occur at the cellular level, leading to reduced plant productivity. However, the foliar application of kaolin (KL) can mitigate the impact of abiotic stress by decreasing leaf temperature and enhancing antioxidant defence. Hence, this study hypothesised that KL-treated grapevine plants growing in NE Portugal would reveal, under summer stressful growing conditions, higher progression and stability of the leaf mitotic cell cycle than the untreated (control) plants. KL was applied after veraison for two years. Leaves, sampled 3 and 5 weeks later, were cytogenetically, molecularly, and biochemically analysed. Globally, integrating these multidisciplinary data confirmed the decreased leaf temperature and enhanced antioxidant defence of the KL-treated plants, accompanied by an improved regularity and completion of the leaf cell cycle relative to the control plants. Nevertheless, the KL efficacy was significantly influenced by the sampling date and/or variety. In sum, the achieved results confirmed the hypothesis initially proposed.

## 1. Introduction

Due to its high economic importance, grapevine (*Vitis vinifera* L.) is one of the most widespread cultivated species. In 2023, the worldwide vineyard’s surface area was estimated to be ca. 7.21 million ha, and the production of wine grapes to be 30.9 million tonnes [1]. Europe and the Mediterranean basin have the world’s most significant vineyard areas and wine production rates [2]. In Portugal, for 2023, a vineyard’s surface area of 182,260 ha and a national wine production of 7.5 million hectolitres were registered [3]. A high number of grapevine varieties cultivated in Portugal can produce wine [4]. The Portuguese autochthonous varieties, “Touriga Franca” (PRT52205) and “Touriga Nacional” (PRT52206), integrate this long list and are the second and the third most cultivated varieties, respectively, in Portugal [4,5,6]. Both “Touriga Nacional” (TN) and “Touriga Franca” (TF) comprise 7% of the Portuguese vineyard area [5,6]. In the “Douro Demarcated Region”, which has belonged to the World Heritage List of UNESCO since 2000, TF is the most cultivated variety, and TN occupies the fourth position [6]. These two grapevine varieties are officially recommended for red and Port wine production in the “Douro Demarcated Region” [7].

TN presents an excellent adaptation to different viticulture ecosystems, being cultivated from north to south on the Portuguese continent and at Azores [6]. TN has high vigour, high fertility, medium-to-high yield, and is susceptible to heat and water stress [6]. TF is traditionally cultivated in the “Douro” region and is supposed to result from a natural cross between “Marufo” and TN varieties [5]. TF has medium-to-high vigour, low-to-medium fertility, high and regular productivity, and high resilience to abiotic factors [5].

Climate change has affected grapevine productivity and wine quality, particularly in narrow geographic regions with microclimates, such as the “Douro Superior” subregion (NE Portugal) of the “Douro Demarcated Region”. During the summer, grapevine varieties such as TF and TN growing in the “Douro Superior” subregion experience a combination of stressful factors, such as high-temperature solar radiation and water deficit [8].

Different abiotic stresses can generate an uncontrolled production and accumulation of reactive oxygen species (ROS) that oxidise lipids, proteins, and nucleic acids and interact with other reactive species like the reactive nitrogen species (RNS), reactive sulphur species (RSS), and reactive carbonyl species (RCS) [9,10,11,12,13,14]. Nevertheless, activating defence mechanisms such as synthesising radical-scavenging compounds under stressful environments improves the plant’s resilience [9,10,11,12,13,14]. Depending on the efficacy of the plant antioxidant system, oxidative stress and, ultimately, cell death may occur [9,10,11,12,13,14]. The failure or delay of the DNA repair mechanisms caused by oxidative stress can lead to structural rearrangements and mitotic anomalies [9,10,11,12,13,14]. Subsequent changes in enzymatic activity and gene regulation may affect plant growth, development, and productivity due to a decrease in photosynthetic activity and the quality of the end products.

For disease control and mitigation of the negative impacts caused by climate change, a short-term measure that consists of the foliar application of an inert particle film of aluminum silicate, commonly named kaolin (KL), has been used in various crops, including grapevine, growing in areas with a Mediterranean-like climate [15,16,17,18]. These authors reported that the KL treatment reduces the surface temperature of the grapevine leaves by reflecting excessive ultraviolet and infrared radiation, improving the photosynthetic activity and antioxidant defence. Despite the knowledge of the physiological and biochemical benefits of KL treatment in grapevine [15,16,17,18], as far as we know, its effects on the leaf mitotic cell cycle have yet to be studied.

Under natural growing conditions, the leaf is the growing organ more exposed to heat and solar radiation [19]. Excessing these abiotic factors may compromise the photosynthetic capacity, mainly in C3 plants such as grapevine [20]. The study of grapevine leaves is critical since these growing organs can evidence the plant status regarding nutrition, vigour, and stress susceptibility [21]. Leaves can be targeted for different and cost-effective analyses, providing faster and more reliable information about plant resilience and productivity [19,21].

Mesophyll cells are the most common type of cell in leaves, and their mitotic activity is restricted to the basal region between the blade and the petiole [22,23,24]. The leaf meristematic or proliferation region (considered an intercalary meristem) maintains a constant size over a limited period during which mesophyll, initial veins, stomata, blade, and petiole cells are produced [23,24]. Then, the cell proliferation stops, and a switch to cell differentiation occurs in the developing leaves [23,24]. However, as verified under controlled conditions, abiotic stress such as heat can impair the grapevine leaf cell division [13], affecting the leaf growth and decreasing the photosynthetic area and, consequently, the vine’s productivity. The synthesis of molecular chaperones or heat shock proteins (HSPs) and heat shock transcription factors (HSFs) is induced in response to heat stress. It represents a crucial element of acquired thermotolerance in all organisms [25].

As verified under open-field and controlled conditions, grapevine varieties may exhibit a differential tolerance to individual or combined abiotic stress factors [15,16,17,18,19,21,26,27,28,29,30]. Plants more tolerant to abiotic stresses can maintain growth and reproductive success by activating cellular, physiological, molecular, and biochemical mechanisms [20], and the resulting changes improve the adaptive potential [31]. The resulting modifications required for plant adaptation include morphological and physiological alterations driven by the continuous activity of meristems [31]. Nevertheless, the meristem activity is modulated by intra- and extracellular stimuli to ensure the balance between cell division and cell differentiation [31]. Cell cycle regulation is essential for plant growth and development [32].

DNA damage from nucleic acids, lipids, and proteins and the failure of the mechanisms for DNA repair of strand breaks caused by stress induces structural rearrangements and/or mitotic spindle irregularities [33]. These anomalies trigger the initiation of cell cycle checkpoints, delaying mitosis and, consequently, plant growth and development [22,34], resulting in yield reduction [35]. The cyclin-dependent kinases (CDKs), a family of serine/threonine kinases, control cell division by regulating the cell cycle progression through its involvement in signalling cascades, triggering adaptive responses to environmental cues [31]. The CDKs are activated by different types of cyclins expressed between transition phases of the cell cycle (G1/S and G2/M) and control DNA replication and mitosis [32,36,37,38]. Still, their activity can be negatively regulated by binding specific inhibitory proteins (INHIBITOR OF CDK/KIP-RELATED PROTEIN, ICK), among other mechanisms [32,36,39]. The expression of *ICK* and other CDK/cyclin inhibitor genes is consistent with a regulatory role of the cell cycle during plant development and response to environmental stimuli that induce cell cycle arresting and delay of response to intra- and extracellular stimuli [38,40,41]. For instance, the *KRP1* gene, a CDK inhibitor, was up-regulated by abscisic acid (ABA), a plant hormone involved in stress-induced cell cycle arresting by repression of the cell cycle genes [38,42,43]. On the other hand, the KL treatment reduces the ABA biosynthesis [44].

Based on previous research and consulted literature, this study hypothesised that KL-treated grapevine leaves growing in the NE of Portugal would present a higher cell cycle progression and more regular mitosis than the untreated (control) leaves under summer’s stressful growing conditions, mainly due to the decrease in leaf temperature and enhancement of the antioxidant defence triggered by KL.

This work aimed to cytogenetically evaluate the regularity and progression of the leaf’s mitotic cell cycle in KL-treated plants of the TF and TN varieties growing under stressful environmental conditions in the summers of 2016 and 2017 and to integrate those data with the expression profiling of two cell cycle regulatory genes (*VvCYCA3* and *VvICK5*), one heat stress-responsive gene (*Hsp17.9A*), and three oxidative stress-responsive genes (*APx*, *CAT* and *MDHAR*) encoding for the antioxidant enzymes APx, CAT, and MDHAR, whose activity was biochemically assayed.

The KL-treated plants showed higher resilience to summer’s stressful growing conditions than the control (untreated) plants, as verified by (i) the down-regulation of the heat-stress responsive gene, confirming the decrease in the leaf temperature; (ii) the down-regulation of the CDK-inhibitor, along with the up-regulation of the A-type cyclin, evidencing cell cycle progression and corroborating the cytogenetic data; and (iii) the global up-regulation of the genes encoding three crucial antioxidant enzymes whose activity was confirmed biochemically. Overall, the present results confirmed the hypothesis initially proposed.

## 2. Materials and Methods

### 2.1. Experimental Site, Kaolin Treatment, and Plant Material

**Figure 1:**
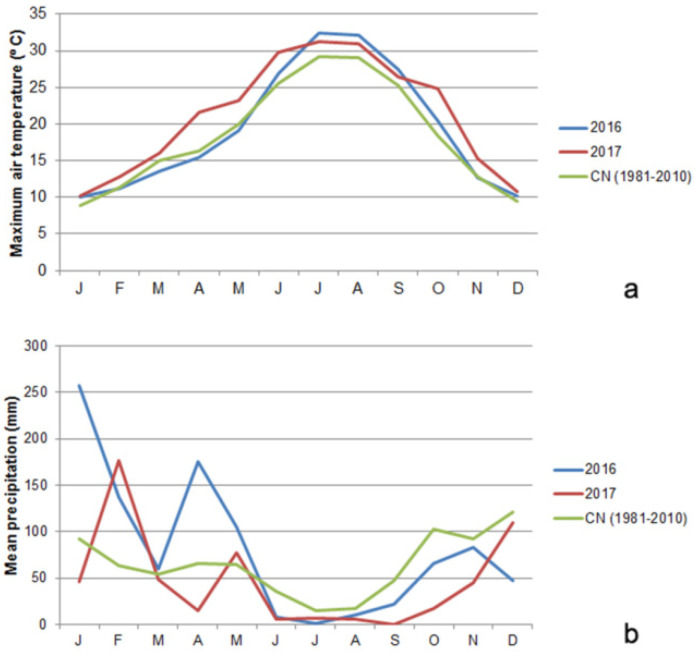
Climatological normal (CN) for the 1981–2010 period, monthly: (**a**) maximum air temperature (°C) and (**b**) mean accumulated precipitation (mm) values registered for the years of 2016 and 2017 by a weather station near the experimental site.

The experimental site was the commercial vineyard of “Quinta do Orgal” (“Quinta do Vallado SA”), located at “Vila Nova de Foz-Côa” (41°05′ N, 7°08′ W; 169 m above sea level) in the “Douro Superior” sub-region (NE Portugal). This vineyard has an east–west orientation on a steep slope (30° N) and a unilateral cordon training system. The TF and TN vines, six years old and grafted onto 110R rootstock, were managed according to commercial organic practices (use of organic fertilisers, non-synthetic pesticides, and spontaneous cover cropping) and deficit irrigated (30% of the reference evapotranspiration) via drip irrigation [17]. The soil of this region is classified as Luvisolic, presenting clay-enriched subsoil [45]. This region presents a warm temperate climate with dry and hot summers and rainfall periods mainly concentrated in winter [17]. These climate features apply to 2016 and 2017, when the KL treatments were performed (Figure 1).

The foliar application of KL on TF and TN plants was conducted after the veraison stage in 2016 and 2017 by spraying an aqueous solution of 5% (*w*/*v*) of KL (WP Surround; Engelhard Corporation, Iselin, NJ, USA) following previous procedures [15,16]. In three vineyard rows, plants of each variety were sprayed uniformly with KL. A second application was performed on the same day to ensure the KL uniformity and adhesion to the leaves. In the other three vineyard rows, plants of each variety were maintained untreated (without KL) to be used as a control.

In 2016 and 2017, three and five weeks after KL application, small and fully expanded leaves were sampled in five plants of each variety (TF and TN) and treatment (KL and control). The leaves were sampled on the 28 July and 22 August 2016 and on the 18 July and 22 August 2017. The summer of 2016 was hotter and drier than 2017 [46,47] (Figure 1). Moreover, two heat waves occurred during the summer of 2017, mainly affecting the Northern region of Portugal; the first occurred at the end of June for two weeks, and the second happened between the 12th and 17th of July for 6–7 days [47].

The sampled leaves were immediately fixed in absolute ethanol and acetic acid in the proportion of 3:1 (*v*/*v*) for cytogenetic analyses or immediately frozen in liquid nitrogen for the gene expression assays. At UTAD, the fixed and frozen leaf samples were stored at −20 °C and −80 °C, respectively.

### 2.2. Cytogenetic Analysis of KL-Treated and Untreated Leaves

Per fixed leaf, an area of 1 cm^2^ of its basal region was cut and digested with an enzymatic solution for the further preparation of cell suspensions of mesophyll dividing cells, following the procedures described by [19]. A volume of 30 µL of each cell suspension was dropped to an ethanol-cleaned glass slide, air-dried in a horizontal position, and aged for 2 h at 60 °C. The mitotic preparations were observed on the phase contrast microscope to confirm the presence of plant material and/or cell scoring. Some mitotic preparations were stained with a drop of mounting medium VectaShield^®^ with DAPI (©Vector Laboratories, Inc., Newark, CA, USA) or an aqueous solution of 100% silver nitrate, as described by [19], to facilitate the cell scoring.

Per preparation, a variable number of interphase and mitotic cells were scored. Mitotic phases and anomalies were identified. Based on these data, the mitotic index (MI) and the percentage of dividing cells with anomalies (DCA), expressed in percentage, were determined with Equations (1) and (2), respectively.

(1) $$\mathrm{MI}\;(\%)=\frac{\mathrm{Number}\;\mathrm{of}\;\mathrm{dividing}\;\mathrm{cells}}{\mathrm{Total}\;\mathrm{number}\;\mathrm{of}\;\mathrm{scored}\;\mathrm{cells}}\times 100,$$

(2) $$\mathrm{DCA}\;(\%)=\frac{\mathrm{Number}\;\mathrm{of}\;\mathrm{dividing}\;\mathrm{cells}\;\mathrm{with}\;\mathrm{anomalies}}{\mathrm{Total}\;\mathrm{number}\;\mathrm{of}\;\mathrm{dividing}\;\mathrm{cells}}\times 100$$

In Equation (1), the total number of scored cells refers to the sum of interphase and mitotic (dividing) cells.

Images were captured with an XC10 charge-coupled device (CCD) digital camera (Olympus America, Inc., Hauppauge, NY, USA) coupled to a microscope Olympus BX41 (Olympus America, Inc., Hauppauge, NY, USA) using the cellSens software (Olympus Soft Imaging Solutions GmbH, Münster, Germany).

### 2.3. Molecular Analysis of KL-Treated and Untreated Leaves

For the quantitative real-time PCR (qPCR) procedures, the minimum information for publication of quantitative real-time PCR experiments (MIQE) guidelines [48] were followed.

Frozen leaves (−80 °C) of both TF and TN varieties and treatments (control and KL) sampled in July and August 2016, and 2017 were quickly ground to a fine powder in liquid nitrogen. Per sample, 250 mg of grounded frozen leaf material was used for total RNA isolation using the CTAB-based method described by [49]. The total RNA samples were purified using the PureLink^TM^ RNA Mini Kit (Ambion^®^, Life Technologies^TM^, USA). The purified total RNA samples were quantified in the spectrophotometer Nanodrop™ ND-1000 (Thermo Fisher Scientific, Inc., Waltham, MA, USA). A total of 3 µL of each total RNA sample was denatured at 65 °C for 10 min in an equal volume of formamide mixed with bromophenol blue and then incubated on ice for 5 min. The integrity of the denatured RNA samples was evaluated after electrophoresis on 2% (*w*/*v*) agarose gels prepared with 1× Tris-borate-EDTA (TBE) buffer under a constant voltage of 100 V and staining with ethidium bromide.

To synthesise complementary DNA (cDNA), 200 ng of each integer and pure total RNA sample, and the High Capacity cDNA Reverse Transcription Kit (Applied Biosystems, Foster City, CA, USA) were used. For the individual amplification of each reference and target gene by qPCR (final volume of 10 µL), 40 ng µL^−1^ cDNA, 1× conc. iQ5TM SYBR Green Supermix Kit (Bio-Rad, Laboratories, Inc., Hercules, CA, USA), and 0.3 µM of forward and reverse primers (Table S1) were used. The primers were ordered from STAB Vida, Portugal.

Negative controls for each gene were included in each 96-well transparent plate, which was sealed with adhesives. Standard curves based on a 10×-dilution series of pooled cDNA samples were performed for each primer pair to determine each gene’s PCR amplification efficiency (E) according to Equation (3) (Figure S1).

[E = 10^(−1/slope)^ − 1] (3)

The qPCR assays were performed using the Stratagene Mx3005P qPCR system (Agilent Technologies, Santa Clara, CA, USA). The cDNA amplification conditions consisted of 95 °C for 3 min, followed by 40 cycles of 95 °C for 15 s, 60 °C for 15 s, and 72 °C for 30 s. After a final extension at 72 °C for 3 min, the samples were held at 55 °C for 3 min then heated to 95 ° C for 1 min with a ramp of 0.5 °C/s to produce the dissociation (melting) curve of the amplified products for validation of the specificity of the amplicons (Figure S2). The amplified products were also run on 2% agarose gels to confirm the presence of a single amplicon (Figure S2) with the expected size (Table S1)

As reference (or housekeeping) genes, the *Vacuolar ATPase subunit G* (*VAG*) and the *Ubiquitin-conjugating enzyme* (*UBC*) genes were used (Table S1). The primers used for the amplification of the *VAG* and *UBC* genes (Table S1) were designed by other authors [50]. The expression of the following target genes was evaluated: (i) *Class II heat shock protein*, *HSP17.9A*; (ii) *A-type cyclin*, *VvCYCA3*; (iii) *CDK-inhibitor*, *VvICK5*; (iv) *Ascorbate peroxidase 1*, *APx1*; (v) *Catalase*, *CAT*; and (vi) *Monodehydroascorbate reductase*, *MDHAR* (Table S1). For the amplification of the six target genes, oligonucleotides (Table S1) earlier designed by other authors [28,38,41] were used. 

Three biological replicates (corresponding to three different plants) and three technical replicates were performed for each qPCR experiment. Per reference and target gene, the two nearest quantification cycle (Cq) values of the biological and technical replicates were chosen to determine the average Cq values (Figure S3). The mean Cq value of each target gene achieved per variety × kaolin treatment × sampling date interaction was normalised to the geometric mean of the Cq values of the two reference genes, *VAG* and *UBC* (Figure S3). The relative expression ratio of each target gene was determined by Equation (4) [51].

(4) $$\mathrm{Relative}\;\mathrm{expression}\;\mathrm{ratio}=\frac{{\left(E\;\mathrm{target}\right)}^{∆\mathrm{Cq}\;\mathrm{target}\;(\mathrm{control}-\mathrm{sample})}}{{\left(E\;\mathrm{reference}\right)}^{∆\mathrm{Cq}\;\mathrm{reference}\;(\mathrm{control}-\mathrm{sample})}}$$

In Equation (4), the E target corresponds to the efficiency amplification value of each target gene (Figure S1; Table S1). The E reference consists of the geometric mean of the efficiency values of the two reference genes (Figure S1). The ∆Cq represents the difference between the Cq values achieved in the control and KL-treated plants for each target or reference gene. Values of relative expression ratio above 1 to infinity were considered up-regulation, and values between 0 and 1 were considered down-regulation [52].

### 2.4. Biochemical Analysis of KL-Treated and Untreated Leaves

Antioxidant enzymes were extracted according to [17]. In brief, lyophilised samples were homogenised using an Ultra Turrax RZR1 (Heidolph, Schwabach, Germany) at 20,000 rpm and 4 °C. The extraction buffer comprised 0.2 M Tris.HCl (pH 8.0), 5 mM dithiothreitol (DTT), 0.5 mM phenylmethylsulfonyl fluoride (PMSF), 10% (*w*/*v*) glycerol, 0.25% (*w*/*v*) Triton X–100, and 2% (*w*/*v*) insoluble polyvinylpolypyrrolidone (PVPP). After centrifuging at 40,000× *g* for 30 min, the supernatants were portioned and stored at −80 °C. The Bradford method [53] was used to determine the protein content. Before the ascorbate peroxidase (APx) enzymatic assay, 1 mM of ascorbic acid (AsA) was added to the protein extract. All enzymatic assays were conducted with saturating substrate concentrations to ensure maximal velocity determination.

The catalase (CAT) (EC 1.11.1.6) activity was assessed by measuring the reduction of H_2_O_2_ at 240 nm for 2 min based on the method outlined by [54] (with an extinction coefficient of 0.0436 mM^−1^ cm^−1^).

The ascorbate peroxidase (APx) (EC 1.11.1.11) activity was determined by observing the decline in absorbance at 290 nm over 2 min, as described by [55] (with an extinction coefficient of 2.8 mM^−1^ cm^−1^).

The monodehydroascorbate reductase (MDHAR) (EC 1.6.5.4) activity was assayed by the decrease in absorbance at 340 nm due to NADH oxidation [55] in a 1 mL reaction mixture containing 50 mM Tris-HCl (pH 7.5), 0.2 mM NADH, 2.5 mM AsA, 2 units ascorbate oxidase, and the enzyme sample.

### 2.5. Statistical Analysis

The cytogenetic and biochemical results are presented as mean ± standard error (S.E.) values per variety, treatment, and sampling date interaction. In these assays, the mean values resulted from three replicates (n = 3) that were simultaneously biological and technical replicates.

The qPCR results are presented as expression ratio per target gene in the KL-treated plants relative to the control (untreated) plants. The relative expression ratio values were calculated based on the mean Cq values of two biological and technical replicates (n = 2), whose standard deviation was lower than 0.5, using the calculation method of Livak and Schmittgen [52].

The analyses of variance (ANOVA), the post hoc Fisher’s protected least significant difference (PLSD) test and the equality of variances F test were performed with the software Statview 5.0 (SAS Institute, Inc., Copyright © 1992–1998, Cary, NC, USA). A pairwise comparison of mean values was also performed with the Tukey test. The *p*-value significance of all statistical analyses was set for probabilities lower than 5% (*p* < 0.05) and 0.1% (*p* < 0.001).

## 3. Results

### 3.1. Cytogenetic Evaluation of the Regularity of the Leaf Mitotic Cell Cycle

**Figure 2:**
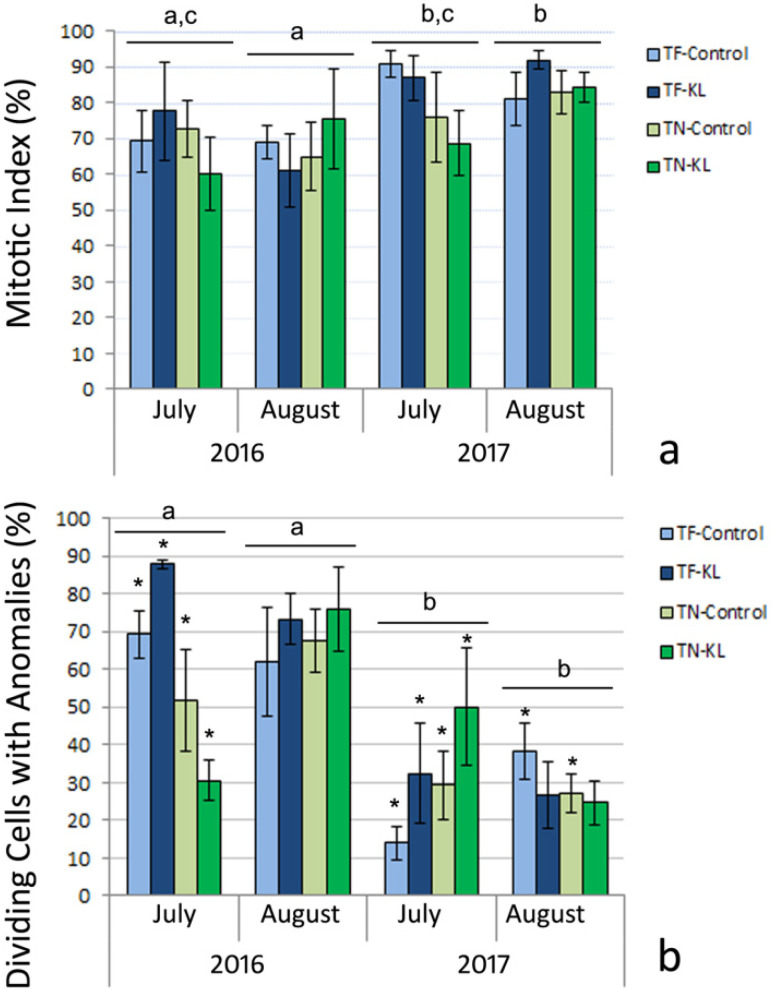
Mean ± standard error (SE) percentage values (resulting from three mitotic preparations that were simultaneously biological and technical replicates; n = 3) of mitotic index (**a**) and dividing cells with anomalies (**b**) determined for the interaction of the individual factors: variety (TF and TN), treatment (control and KL), and sampling date (July and August of 2016, and July and August of 2017). Different lowercase letters represent statistically significant differences (*p* < 0.05 in (**a**) and *p* < 0.001 in (**b**)) among sampling dates. (*)—Statistically significant differences (*p* < 0.05) between varieties per treatment and sampling date.

The cytogenetic parameters, MI and DCA, were determined based on the scoring of interphase and mitotic cells. Figure 2 presents the mean values of MI and DCA for the interaction among the individual factors: variety (TF and TN), treatment (KL and control), and sampling date (July and August 2016 and July and August 2017).

Concerning the cytogenetic parameter MI, statistically significant differences (*p* < 0.05) were detected among the sampling dates concretely between July 2016 and August 2017, August 2016 and August 2017, and August 2016 and July 2017 (Figure 2a).

TF plants treated with KL in July 2016 and August 2017 and TN plants treated with KL in August 2016 and August 2017 showed higher average values of MI than the respective control plants (Figure 2a). Nonetheless, no statistical significance (*p* > 0.05) between varieties, treatments, or their interaction was found. Globally, the mean MI values were high in all interactions presented in Figure 2a. However, it should be considered that MI calculation (Equation (1)) accounts for all (normal and irregular) mitotic cells.

Regarding the cytogenetic parameter DCA, statistically significant differences (*p* < 0.001) were detected among the sampling dates, namely, between July 2016 and July 2017, July 2016 and August 2017, August 2016 and July 2017, and August 2016 and August 2017 (Figure 2b). Statistically significant differences (*p* < 0.05) were also found for the variety × sampling date interaction (Figure 2b). Generally, the highest mean DCA values were determined for the two sampling dates in 2016 (Figure 2b). In July 2016, the TN plants treated with KL presented a mean DCA value lower than that of the control plants (Figure 2b). In August 2017, the leaves of both varieties collected in KL-treated plants showed mean DCA values lower than those found in the respective control plants (Figure 2b).

**Figure 3:**
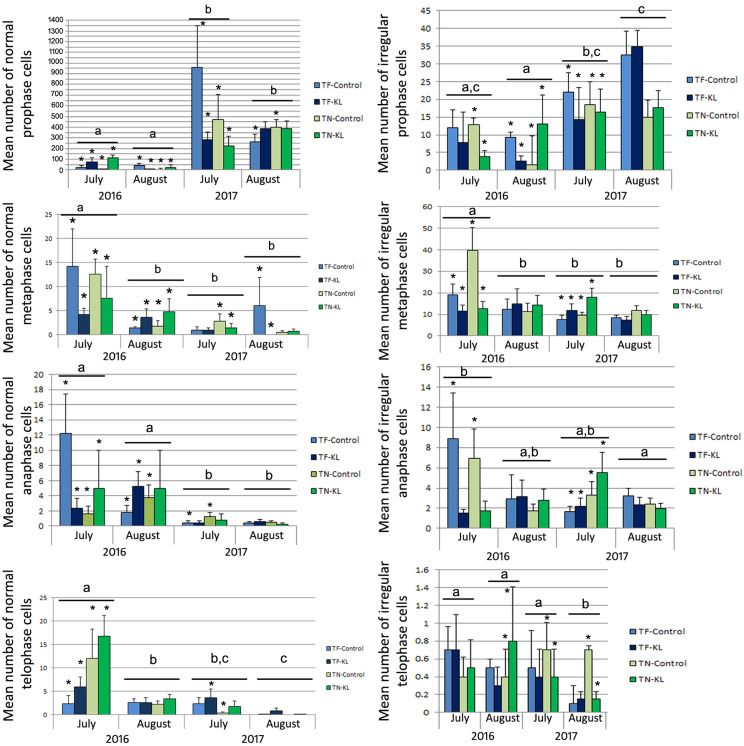
Mean ± standard error (SE) values (resulting from three mitotic preparations that were simultaneously biological and technical replicates; n = 3) of normal (left column) and irregular (right column) dividing cells determined for the interaction of the individual factors: variety (TF and TN), treatment (control and KL), and sampling date (July and August of 2016 and July and August of 2017). Different lowercase letters represent statistically significant differences (*p* < 0.05) among sampling dates. (*)—Statistically significant differences (*p* < 0.05) between varieties and/or treatments per sampling date.

Figure 3 presents the mean number of normal and irregular mitotic cells in prophase, metaphase, anaphase, and telophase determined for the variety × treatment × sampling date interaction.

Most normal dividing cells were in prophase (Figure 3, left column). The highest mean numbers of normal prophase cells were registered in the control plants of both varieties in July 2017 (Figure 3, left column). In fact, for both varieties, the lowest average values of normal metaphase, anaphase, and telophase cells were registered in July and August of 2017 (Figure 3, left column). The control plants of the TF variety showed the highest average values of normal metaphase and anaphase cells, but the lowest average of normal telophase cells in July 2016 (Figure 3, left column). On the other hand, the highest mean values of normal telophase cells in 2016 and 2017 were registered in KL-treated plants of TN and TF, respectively (Figure 3, left column), suggesting completion of the leaf mitotic cell cycle.

**Figure 4:**
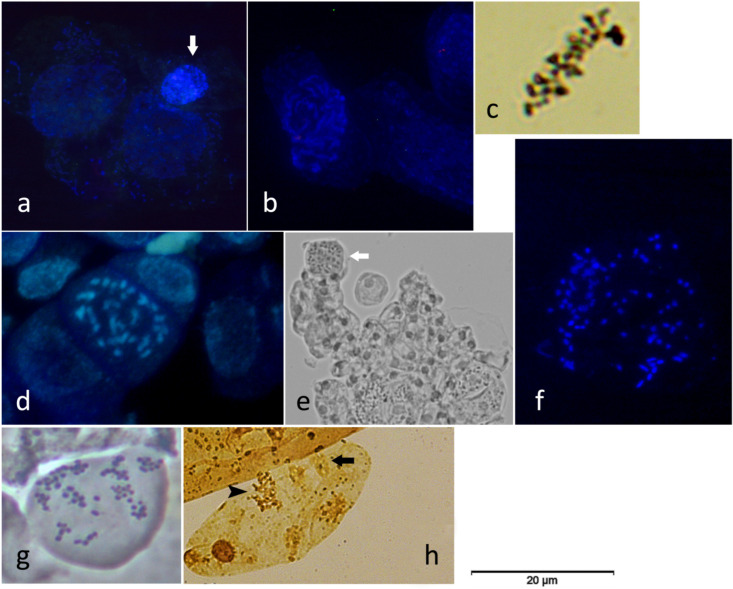
Normal (**a**–**c**) and irregular (**d**–**h**) dividing cells of grapevine captured in the fluorescence microscope after DAPI staining (**a**,**b**,**d**,**f**), on the optical microscope upon silver nitrate staining (**c**,**h**), or on the phase contrast microscope (**e**,**g**) during cell scoring: (**a**) two normal prophase cells and one irregular prophase showing chromatin stickiness (arrow); (**b**) late prophase (normal cell); (**c**) normal metaphase; (**d**,**e**) C-mitoses or C-metaphases, indicated with an arrow in (**e**); (**f**) late anaphase with disturbed chromosomal orientation and laggard chromosomes; (**g**) multipolar anaphase; (**h**) telophase with chromatin stickiness (arrow) and one C-mitosis (arrowhead).

The control plants of the TN variety showed the highest mean value of irregular metaphase cells in July 2016, followed by the high average of irregular prophases detected in the control and KL-treated TF plants in August and July 2017 (Figure 3, right column). Moreover, the mean numbers of irregular anaphase and telophase cells of both varieties registered in 2016 and 2017 were higher than those determined for the normal mitotic phases (Figure 3). The most common mitotic anomalies observed during this work are presented in Figure 4.

For each normal mitotic phase, the mean number of dividing cells presented statistically significant differences (*p* < 0.001, except for metaphase, whose *p*-value was lower than 0.05) among the sampling dates. The mean number of normal anaphase cells revealed statistical significance (*p* < 0.05) for the variety × treatment × sampling date interaction, whereas the normal telophase cells showed significant differences (*p* < 0.05) between treatments.

The mean number of irregular prophase, metaphase, and anaphase cells showed statistically significant differences (*p* < 0.05) between varieties, treatments, among sampling dates and/or for the sampling date × variety interaction.

Overall, among the individual factors analysed, the sampling date had the most relevant effect on the regularity and progression of the leaf mitotic cell cycle.

### 3.2. Regulation of the Leaf Mitotic Cell Cycle Assayed by qPCR

To complement the cytogenetic analysis of the leaf cell division, the expression profiling of the target genes *VvCYCA3* and *VvICK5*, involved in cell cycle regulation, was assayed by qPCR.

**Figure 5:**
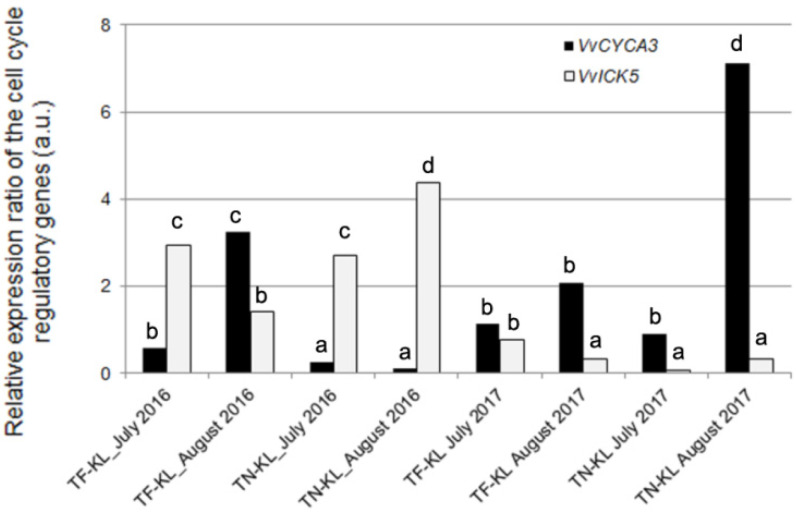
Expression ratio of the cell cycle regulatory genes, *VvCYCA3* and *VvICK5*, determined per variety × kaolin treatment × sampling date interaction, relative to the respective control (untreated) plants. Different lowercase letters represent statistically significant differences (*p* < 0.05) among interactions per target gene.

Figure 5 presents the expression ratio of the *VvCYCA3* and *VvICK5* genes per variety × kaolin × sampling date interaction relative to the respective control (untreated) plants.

According to the assumptions of Livak and Schmittgen [52], the *VvICK5* gene showed up-regulation in all variety × kaolin treatment × sampling dates of 2016 and, contrastingly, down-regulation in all variety × kaolin treatment × sampling dates of 2017 (Figure 5). The *VvCYCA3* gene presented up-regulation in 50% of the variety × kaolin treatment × sampling date interactions, mainly in August 2017 for both varieties (Figure 5). The variety × kaolin treatment × sampling date interactions that showed an up-regulation of the *VvCYCA3* gene and a down-regulation of the *VvICK5* gene suggest the mitotic cell cycle progression under the stressful growing conditions during the summer.

These molecular data achieved with the cell cycle regulatory genes corroborated the cytogenetic results in Figure 3.

### 3.3. Expression Profile of the Hsp17.9A Gene

**Figure 6:**
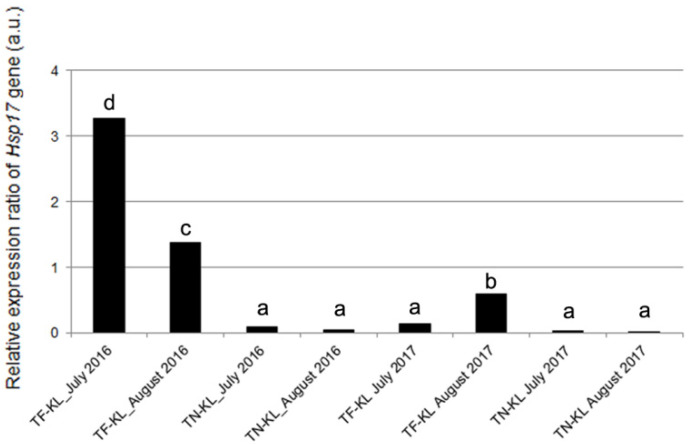
Expression ratio of the *Hsp17.9A* gene determined per variety × kaolin treatment × sampling date interaction relative to the respective control (untreated) plants. Different lowercase letters represent statistically significant differences (*p* < 0.05) among the different interactions.

Heat stress might halt cell division, but the foliar application of KL, which decreases the leaf surface temperature, may allow the cell cycle to progress. Therefore, in this work, the expression of the gene encoding for the small heat shock protein, *Hsp17.9A*, was analysed in the different variety × kaolin treatment × sampling date interactions and compared to the respective control plants (Figure 6).

Most variety × kaolin treatment × sampling date interactions showed down-regulation of the *Hsp17.9A* gene relative to the control (untreated) plants. The two exceptions were found in July and August 2016 for the TF variety, which showed a significant up-regulation of the *Hsp17.9A* gene (Figure 6).

### 3.4. Molecular and Biochemical Analyses Related to the Antioxidant Response

**Figure 7:**
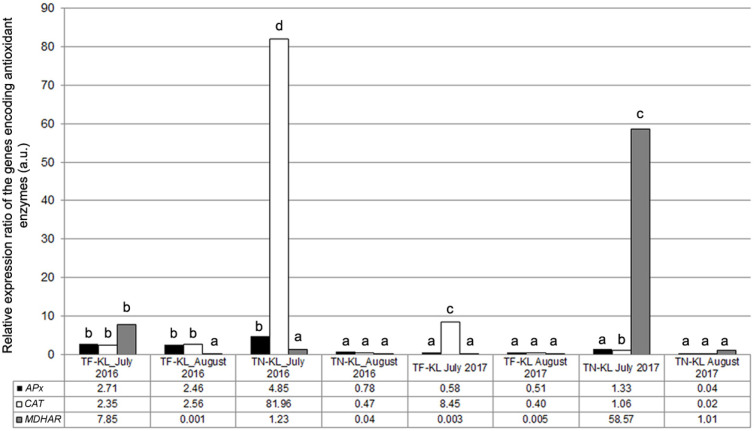
Expression ratio of the genes encoding for the synthesis of the APx, CAT, and MDHAR antioxidant enzymes, determined per variety × kaolin treatment × sampling date interaction, relative to the respective control (untreated) plants. Different lowercase letters represent statistically significant differences (*p* < 0.05) among interactions per target gene.

To evaluate a putative enhancement of the antioxidant defence in the KL-treated plants relative to the control (untreated) plants, the expression ratio of three genes encoding enzymes crucial to ROS scavenging, APx, CAT, and MDHAR, was determined (Figure 7).

**Figure 8:**
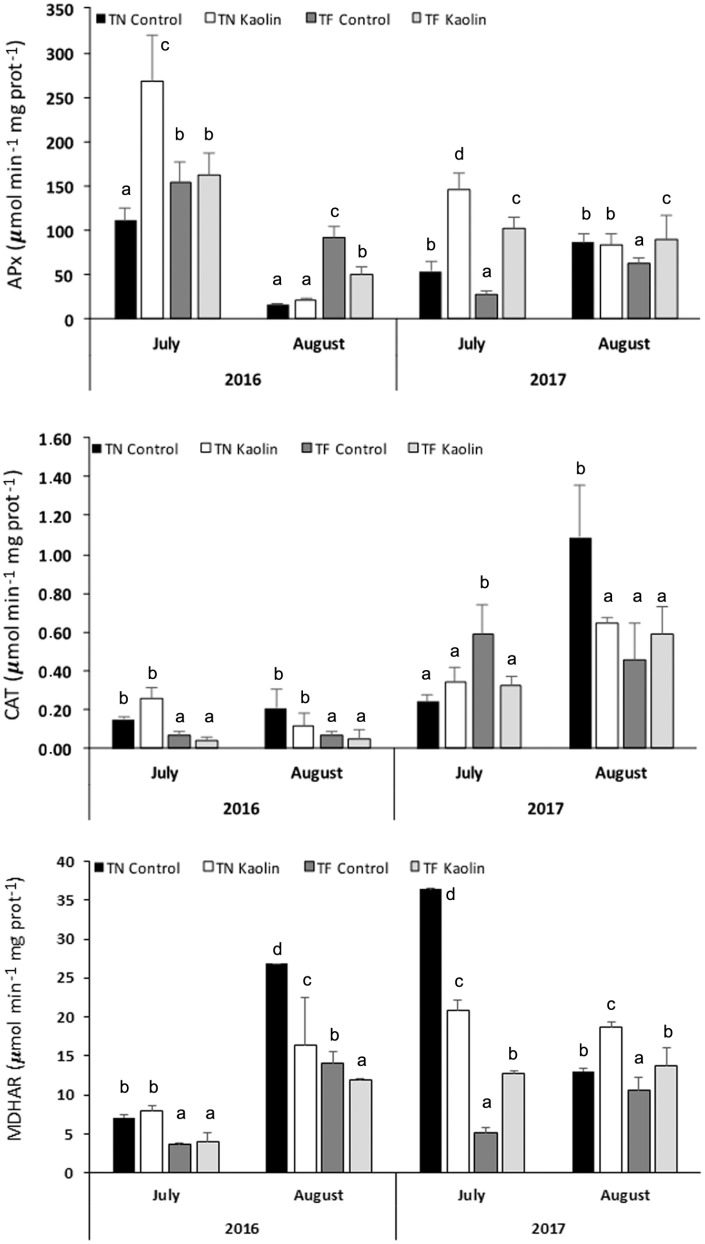
Mean (±standard error, S.E.) values (resulting from n = 3 biological and technical replicates) of enzymatic activity of the APx, CAT, and MDHAR enzymes, determined in leaf extracts of control and KL-treated plants per grapevine variety × treatment × sampling date interaction. The different lowercase letters in each enzyme’s graph represent statistically significant differences (*p* < 0.05) among the variety × treatment interactions per sampling date.

The *APx* gene showed up-regulation in 50% of the variety × kaolin treatment × sampling date interactions, particularly in 2016 (Figure 7). The *CAT* gene was up-regulated in five of the eight analysed variety × kaolin treatment × sampling date interactions and significantly up-regulated in the KL-treated TN plants sampled in July 2016 (Figure 7). The *MDHAR* gene presented up-regulation in three of the eight analysed interactions, namely, in the TN × KL × July 2017 interaction and in the KL-treated plants of both varieties sampled in July 2016 (Figure 7). In August 2017, the three antioxidant genes showed down-regulation in KL-treated plants of both varieties (Figure 7). The enzymatic activity results (Figure 8) partially corroborated the *APx*, *CAT*, and *MDHAR* gene expression profiling (Figure 7).

The activity of the APx enzyme was increased in the KL-treated plants of both varieties in July 2016 and July 2017 and in the KL-treated plants of the TF variety in August 2017, relative to their respective control plants (Figure 8). The highest CAT activities were registered in control plants of both varieties in August and July 2017, followed by KL-treated plants in August 2017 (Figure 8). The highest MDHAR activities were detected in TN control plants sampled in August 2016 and July 2017 (Figure 8). In July 2016, the KL-treated plants of both varieties showed a slight increase in MDHAR activity relative to their control plants (Figure 8). This increase was more pronounced in Kl-treated plants of TF in July 2017 and in KL-treated plants of both varieties in August 2017 (Figure 8).

The biochemical results (Figure 8) were partially coherent with the gene expression profiling of the antioxidant genes (Figure 7), namely, for the APx activity in 2016, whose highest values corresponded to the up-regulation of the *APx* gene in the TF × KL × July 2016, TF × KL × August 2016, and TN × KL × July 2016 interactions. Also, the highest MDHAR activities corresponded to the up-regulation of the *MDHAR* gene in the TF × KL × July 2016, TN × KL × July 2016, TN × KL × July 2017, and TN × KL × August 2017 interactions. The CAT activity (Figure 8) did not present similarities with the up- or down-regulation profiles of the *CAT* gene (Figure 7). Nevertheless, most molecular and biochemical results revealed enhanced antioxidant defence in the KL-treated plants.

## 4. Discussion

There is a growing interest in the in-depth knowledge of the physiological, biochemical, molecular, and cellular mechanisms underlying the plant responses and tolerance to abiotic stress for further testing or development of potential mitigation strategies towards sustainable agricultural production [9]. The most common plant responses to abiotic stress involve changes in photosynthesis, growth, protein synthesis, hormonal metabolism, transcription, signalling networks, and stimulation of the cellular defence machinery [56].

Plant growth is closely connected with the environment; hence, plants decrease their cell division rate under stress to better adapt or wait for more favourable conditions. Considering that the leaf cell cycle, when halted in response to abiotic stress, delays or impairs cell division and cell differentiation, negatively influencing the leaf growth, such reduction of the photosynthetic area implies a decrease in the plant yield [19,31,33]. Cell division regulation under stress was not yet wholly unravelled [31]. Even so, we should be aware that the modulation of cell division could influence the plant’s phenotypic plasticity, adaptation to climate fluctuations, and crop productivity [31].

As widely known from previous studies based on physiological, biochemical, and/or molecular approaches, the application of KL on grapevine leaves decreases the leaf surface temperature, lowers DNA methylation, and boosts the plant’s antioxidant potential [15,16,17,18,57], among other benefits. The heat shock proteins could be up-regulated under different abiotic stresses beyond heat stress [25]. Nonetheless, the down-regulation of the *Hsp17.9A* gene detected in most of the variety × KL × sampling date interactions analysed in the present work, being more pronounced in KL-treated TN plants and sampled in July and August of 2017, confirmed the assumption that this foliar treatment decreases the leaf temperature. This feature prevents the halt of the leaf mitotic cell cycle and DNA damage due to oxidative stress, avoided by the enhanced antioxidant potential assayed molecularly and biochemically. Furthermore, the novelty of this study relies on integrating those physiological, biochemical, and molecular data with the cytogenetic and transcriptional evaluation of the cell cycle regularity and progression whose dates were coherent. The average cell division rate (MI) was high due to the highest number of dividing cells in prophase or metaphase (both normal and irregular), suggesting cell cycle arresting in response to the summer’s stressful environmental conditions. Similar results were previously found in these and other grapevine varieties under different abiotic stresses [19,21]. Beyond this cytogenetic parameter, attention should be paid to the DCA parameter, which was higher globally in the hottest and driest year, 2016 (Figure 1). This result, along with the up-regulation of the *Hsp17* gene detected in July and August 2016, evidenced that under extremely high air temperatures, the foliar application of KL loses effectiveness. The high temperature impairs the microtubules’ structure and/or function, inducing mostly mitotic spindle anomalies, such as C-mitosis, and disturbing chromosomal orientation and cell polarity (Figure 4). Similar cell cycle anomalies were detected in these and other grapevine varieties under stressful abiotic conditions [19,21]. Although the best results of this work were achieved in leaves sampled five weeks after the KL application, recurrent KL applications are suggested in summers with extremely high air temperatures. Nevertheless, the high average MI values coupled with the lowest average DCA values, verified in the summer of 2017, validated the effectiveness of KL in counteracting the halt of leaf cell division under stressful environmental conditions, contributing to leaf growth. Regarding cell cycle progression, the telophase cells presented the lowest average values. This cytogenetic result was confirmed by the expression profiling of the *VvICK5* gene, whose highest relative expression ratio values were detected in all varieties × KL × 2016 interactions. In the summer of 2017, all the analysed interactions showed the highest relative expression ratio of the *VvCYCA3* gene in the KL-treated plants relative to the control group, suggesting cell cycle progression. The cytogenetic and molecular evidence achieved in this work reinforce the usefulness of multidisciplinary and integrative approaches to understanding cell cycle regulation and its response to stressful environmental conditions.

Beyond the known advantages of KL treatment at the physiological and biochemical levels previously reported for the grapevine plant, berry, and wine [15,16,17,18,57], the present work deepened our knowledge of its cellular, molecular, and biochemical effects on the leaves of plants growing under summer’s stressful environmental conditions.

## 5. Conclusions

The global results of this work allowed for the acceptance of the proposed hypothesis by demonstrating that plants from the wine-producing varieties, TF and TN, treated with KL, presented higher regularity and progression of the leaf mitotic cell cycle than the untreated (control) plants. These results were probably due to the decrease in the leaf temperature and enhancement of the antioxidant defence shown by the KL-treated plants. These two benefits of the KL treatment can avoid oxidative stress and consequent DNA damage, which causes mitotic abnormalities and cell cycle arrest, impairing leaf growth and reducing plant productivity. Nevertheless, the KL treatment efficacy was highly influenced by the sampling date. Therefore, during summers with extremely high air temperatures, the recurrent foliar application of KL is recommended.

Overall, the multidisciplinary information gathered by previous and present investigations endorses the KL treatment as a practical and feasible short-term strategy by which to mitigate the negative impacts of stressful environmental conditions occurring during the summer, compatible with sustainable viticulture in the context of climate change.

## Data Availability

The raw data supporting the conclusions of this article will be made available by the authors on request.

## Supplementary Materials

The following supporting information can be downloaded at https://www.mdpi.com/article/10.3390/genes15060747/s1: Figure S1: Standard curves of the reference and target genes; Figure S2: Dissociation curves (a–f) of the six target genes (upper image) and amplicons of the reference and target genes visualised after electrophoresis on 2% agarose gels stained with ethidium bromide (lower image). Notes: M—Molecular weight marker GeneRuler DNA Ladder Mix (Thermo Fisher Scientific). Lanes 1 to 3—*VAG*; lanes 4 to 6—*UBC*; lanes 7 and 8—*Hsp17*; lanes 9 to 11; *VvCYCA3*; lanes 12 to 14: *VvICK5*; lanes 15 to 17: *APx*; lanes 18 to 21: *CAT*; and lane 22: *MDHAR*; Figure S3: (a) Mean Cq (±standard deviation) values resulting from two biological and technical replicates (n = 2) of the reference (*VAG* and *UBC*) and target (*Hsp17*, *VvCYCA3*, *VvICK5*, *APx*, *CAT*, and *MDHAR*) genes and respective (b) mean of normalised ∆Cq (±standard deviation) values, used for the calculation of the relative expression ratio of each target gene per variety × kaolin × sampling date interaction; Table S1: qPCR primers used in this work and respective information.
