# Supplementary material for: Cytogenetic and Molecular Effects of Kaolin’s Foliar Application in Grapevine (Vitis vinifera L.) under Summer’s Stressful Growing Conditions
Source: Genes (Basel). 2024 Jun 6;15(6):747. doi: 10.3390/genes15060747 (PMC11202698; doi:10.3390/genes15060747)
Supplement: Supplementary file 1 [file genes-15-00747-s001.zip › Table S1.pdf]

Table S1. qPCR primers used in this work and respective information.

Reference genes	*NIH - NCBI Reference Sequence	Sequence (5′→3′)	Expected amplicon size (bp)	Reference of the primer sequences	Amplification efficiency (E) determined in this work
Vacuolar ATPase subunit G (*VAG*)	XM_002281110.1	F: TTGCCTGTGTCTCTTGTTC<br>R: TCAATGCTGCCAGAAGTG	174	[50]	98.2%
Ubiquitin conjugating enzyme (*UBC*)	EE253706<br>GenBank: EE253706.1	F: CATAAGGGCTATCAGGAGGAC<br>R: TGGCGGTCGGAGTTAGG	161	[50]	105.6%
**Target genes**	***NIH - NCBI Reference Sequence**	**Sequence (5′→3′)**	**Expected amplicon size (bp)**	**Reference of the primer sequences**	**Amplification efficiency (E) determined in this work**
Class II heat shock protein 17.9 kDa (HSP17.9A)	XM_002280644.4<br>Replaced by:<br>Gene Id: 100268056;<br>LOC 100268056: 17.3 kDa class II heat shock protein *Vitis vinifera*(wine grape)	F: CGTCAAGGAGTACCCCAATTC<br>R: AACTTCCCCACCCTCCTCT	177	[28]	87%
A-type cyclin VvCYCA3	** GENOSCOPE Locus GSVIVT01009399001	F: GATTTTGGTGGATTGGTTGG<br>R: ACCCAATAGCTGGAGCCTCT	150	[38]	109.9%
Cyclin-dependent-kinase (CDK) inhibitor VvICK5	** GENOSCOPE Locus GSVIVT01021078001	F: AAGAGGTGAGGTTGCGGTTA<br>R: GCTAGAGCTTGCCTGCTGTT	150	[38,41]	110.3%
Ascorbate peroxidase 1 (*APX1*), cytosol	XM_002284731.4<br>Replaced by:<br>Gene ID: 100233013;<br>LOC 100233013: L-ascorbate peroxidase 2, cytosolic *Vitis vinifera* (wine grape), also known as: VIT_00025104001, APX1	F: GCCCCACCATTGACTTTGTACC<br>R: ATCGCTCTGGATGTGCCCTTC	218	[28])	106.9%

Table S1. *Continued.*

Reference genes	*NIH - NCBI Reference Sequence	Sequence (5′→3′)	Expected amplicon size (bp)	Reference of the primer sequences	Amplification efficiency (E) determined in this work
Catalase (*CAT*), peroxisome	AF236127<br>GenBank: AF236127.1<br>*Vitis vinifera* catalase (GCat) mRNA, complete cds	F: GTGCAGTCAAACTGTGCCTTAG<br>R: GAGCGAAGAACAGGCTACAGAT	200	[28]	108.5%
Monodehydroascorbate reductase (*MDHAR*), cytosol, peroxisome.	XM_002282964<br>Replaced by: NM_001281042.1<br>*Vitis vinifera* monodehydroascorbate reductase (LOC100233035), mRNA	F: GCCCCACCATTGACTTTGTACC<br>R: ATCGCTCTGGATGTGCCCTTC	140	[28]	107.9%

* NIH – NCBI : National Library of Medicine – National Center for Biotechnology Information. ** Sequences not currently available on the web. GENOSCOPE [http://www.genoscope.cns.fr]

References

[28] Carvalho, L.C.; Coito, J.L.; Colaço, S.; Sangiogo, M.; Amâncio, S. Heat stress in grapevine: the pros and cons of acclimation. *Plant Cell Environ*. **2015a,** *38*, 777–789.

[38] Vergara R, Noriega X, Aravena K, Prieto H and Pérez FJ. ABA Represses the Expression of Cell Cycle Genes and May Modulate the Development of Endodormancy in Grapevine Buds. Front. Plant Sci. **2017**, *8*:812.doi: 10.3389/fpls.2017.00812.

[41] Vergara, R., Noriega, X., Parada, F. et al. Relationship between endodormancy, *FLOWERING LOCUS T* and cell cycle genes in *Vitis vinifera* . *Planta* **2016** , *243*, 411–419.

[50] Borges, A.F.; Fonseca, C.; Ferreira, R.B.; Lourenço, A.M.; Monteiro, S. Reference gene validation for quantitative RT-PCR during biotic and abiotic stresses in *Vitis vinifera*. *PLoS ONE* **2014,** *9(10)*, e111399. doi:10.1371/journal.pone.0111399.
