# Supplementary material for: Cytogenetic and Molecular Effects of Kaolin’s Foliar Application in Grapevine (Vitis vinifera L.) under Summer’s Stressful Growing Conditions
Source: Genes (Basel). 2024 Jun 6;15(6):747. doi: 10.3390/genes15060747 (PMC11202698; doi:10.3390/genes15060747)
Supplement: Supplementary file 1 [file genes-15-00747-s001.zip › Figure S1.pdf]

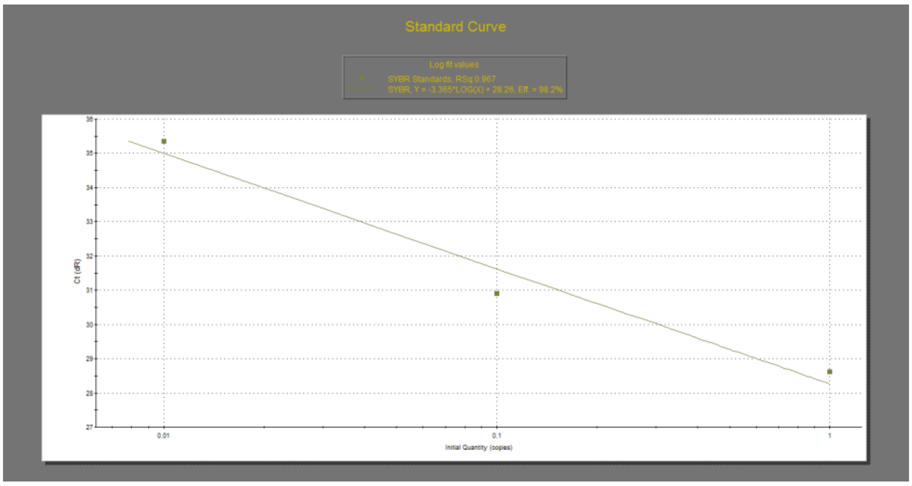

Reference gene: *VAG*

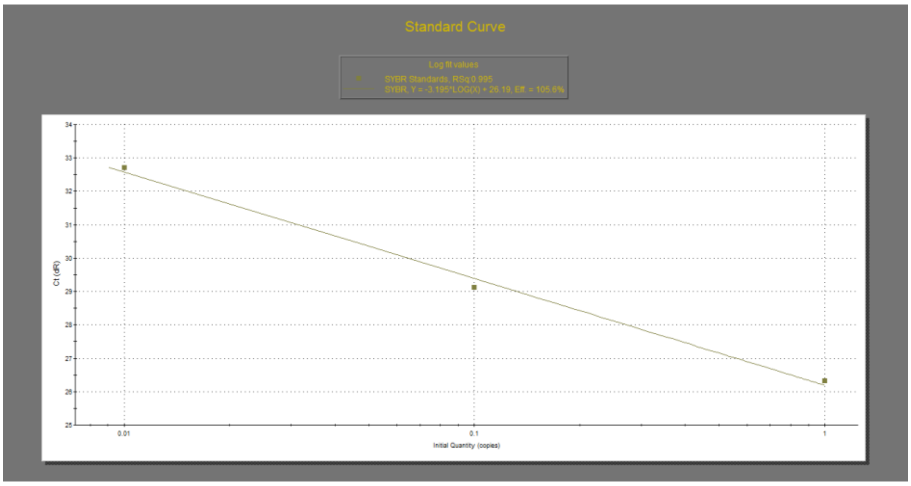

Reference gene: *UBC*

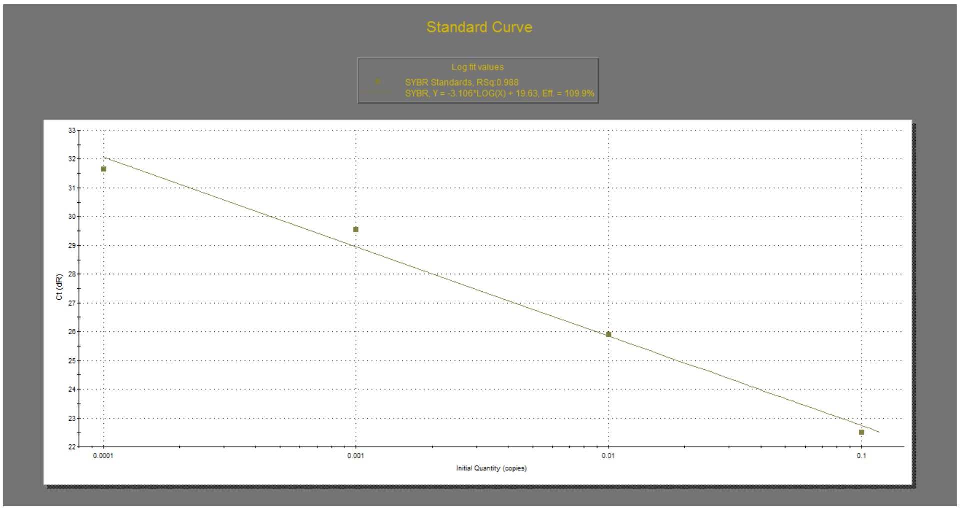

Target gene: *VvCYCA3*

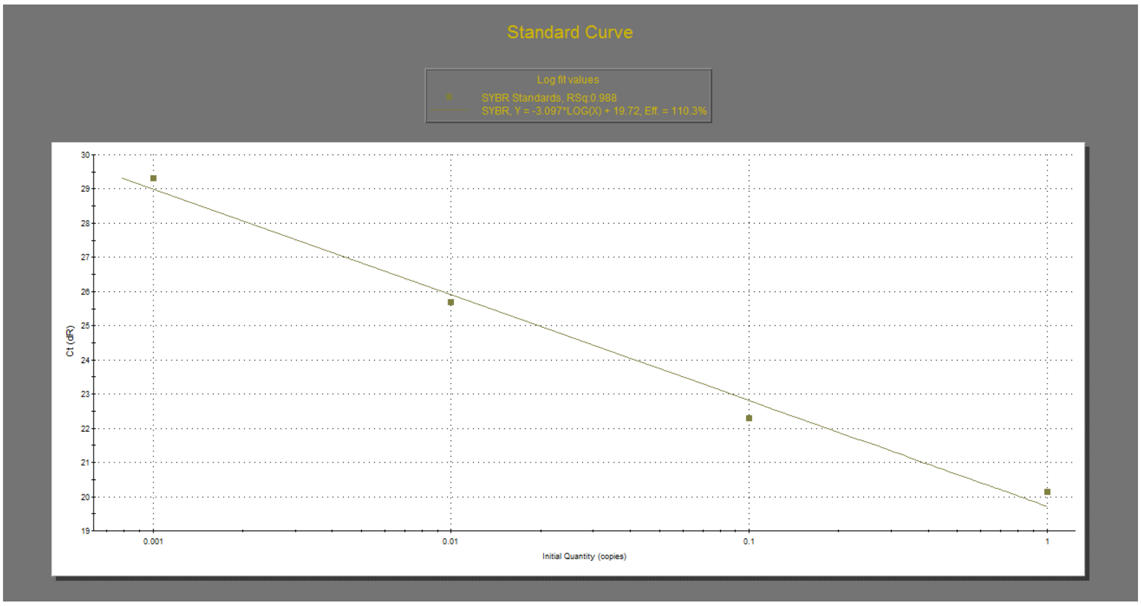

Target gene: *VvICK5*

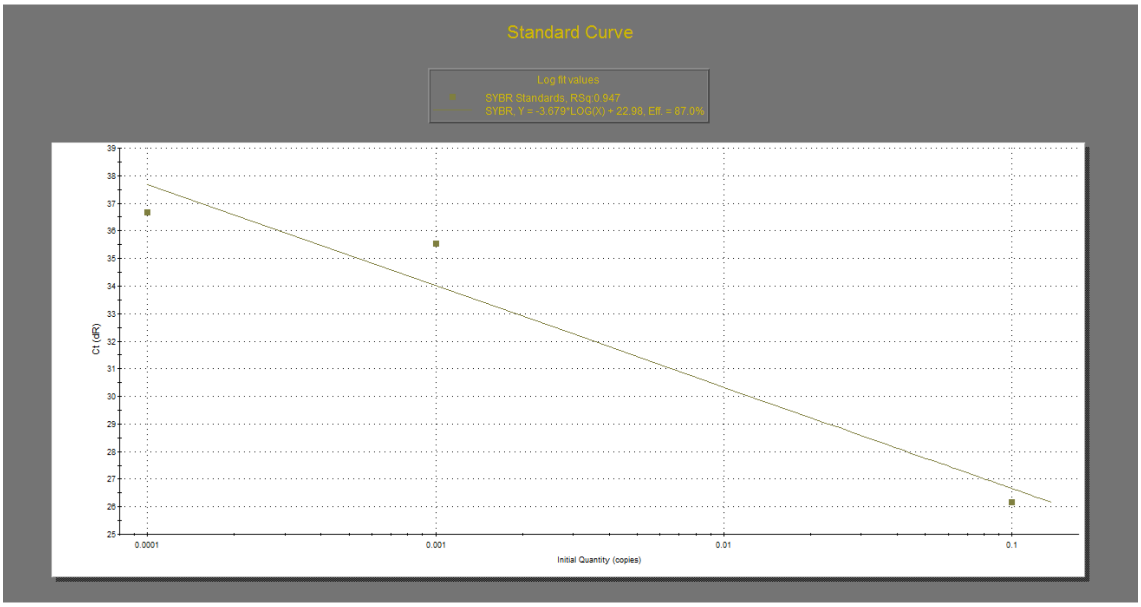

Target gene: *Hsp17.9A*

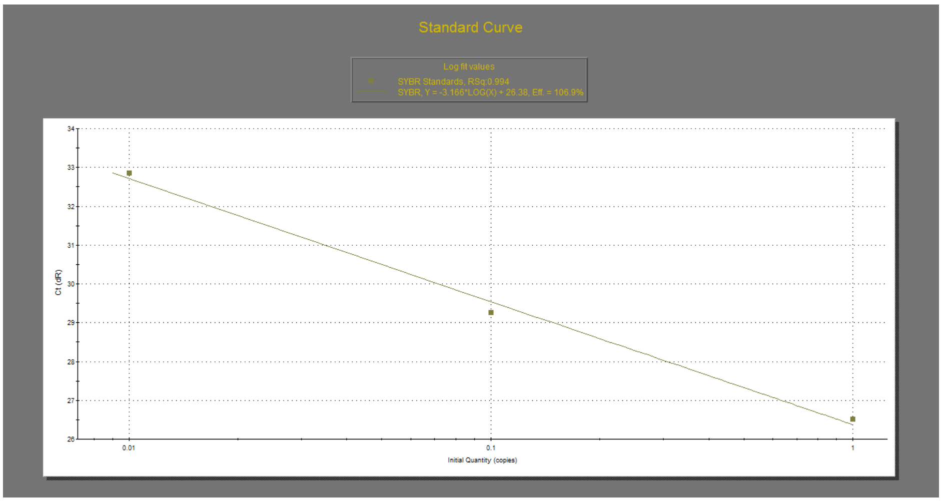

Target gene: *APX1*

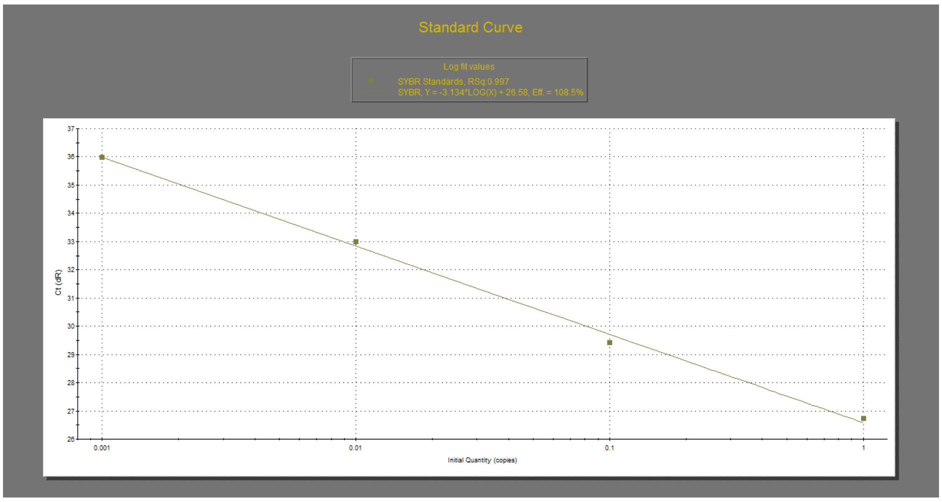

Target gene: *CAT*

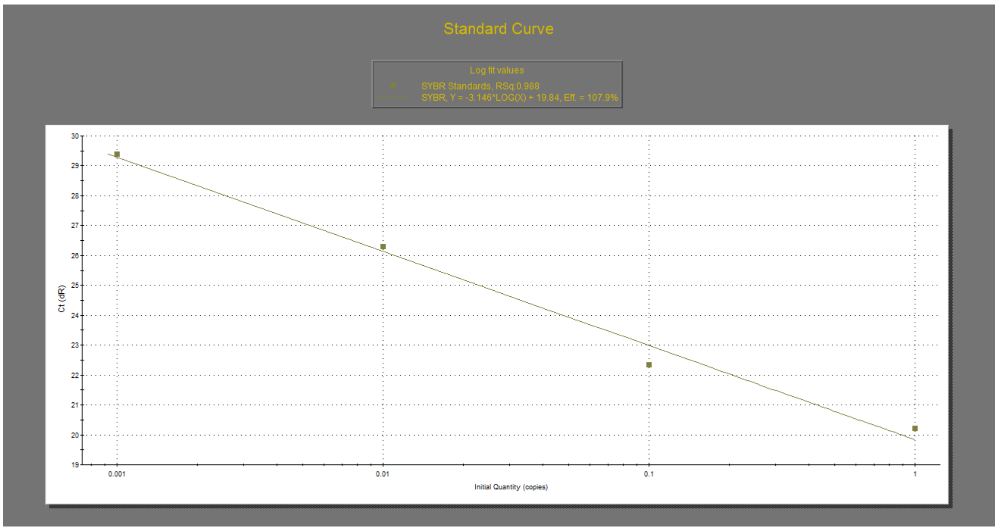

Target gene: *MDHAR*

Figure S1. Standard curves of the reference and target genes.
