# Supplementary material for: Cytogenetic and Molecular Effects of Kaolin’s Foliar Application in Grapevine (Vitis vinifera L.) under Summer’s Stressful Growing Conditions
Source: Genes (Basel). 2024 Jun 6;15(6):747. doi: 10.3390/genes15060747 (PMC11202698; doi:10.3390/genes15060747)
Supplement: Supplementary file 1 [file genes-15-00747-s001.zip › Figure S2.pdf]

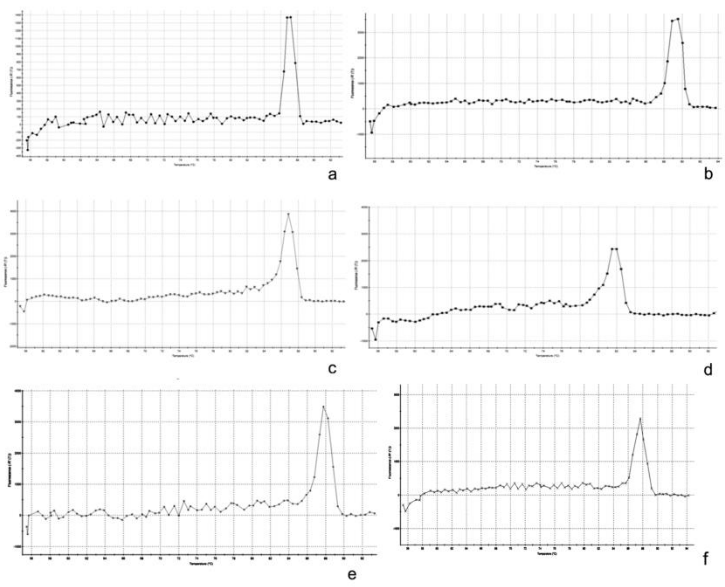

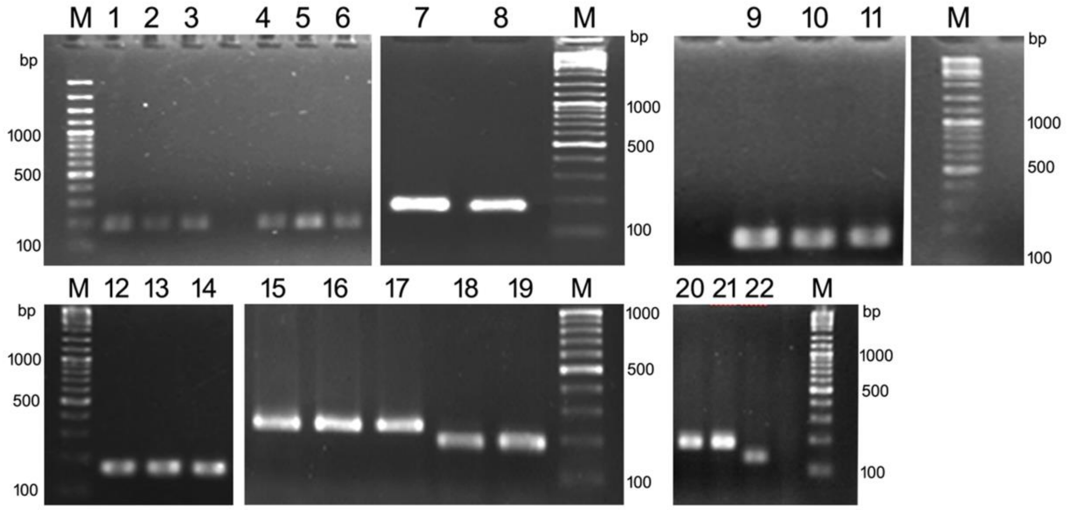

Figure S2. Dissociation curves (a-f) of the six target genes (upper image); and amplicons of the reference and target genes visualized after electrophoresis on 2% agarose gels stained with ethidium bromide (lower image). Notes: M – Molecular weight marker GeneRuler DNA Ladder Mix (Thermo Fisher Scientific). Lanes 1 to 3 - *VAG*; lanes 4 to 6 - *UBC*; lanes 7 and 8 – *Hsp17*; lanes 9 to 11; *VvCYCA3*; lanes 12 to 14: *VvICK5*; lanes 15 to 17: *APx*; lanes 18 to 21: *CAT*; and lane 22: *MDHAR*.
