# Supplementary material for: Cytogenetic and Molecular Effects of Kaolin’s Foliar Application in Grapevine (Vitis vinifera L.) under Summer’s Stressful Growing Conditions
Source: Genes (Basel). 2024 Jun 6;15(6):747. doi: 10.3390/genes15060747 (PMC11202698; doi:10.3390/genes15060747)
Supplement: Supplementary file 1 [file genes-15-00747-s001.zip › Figure S3.pdf]

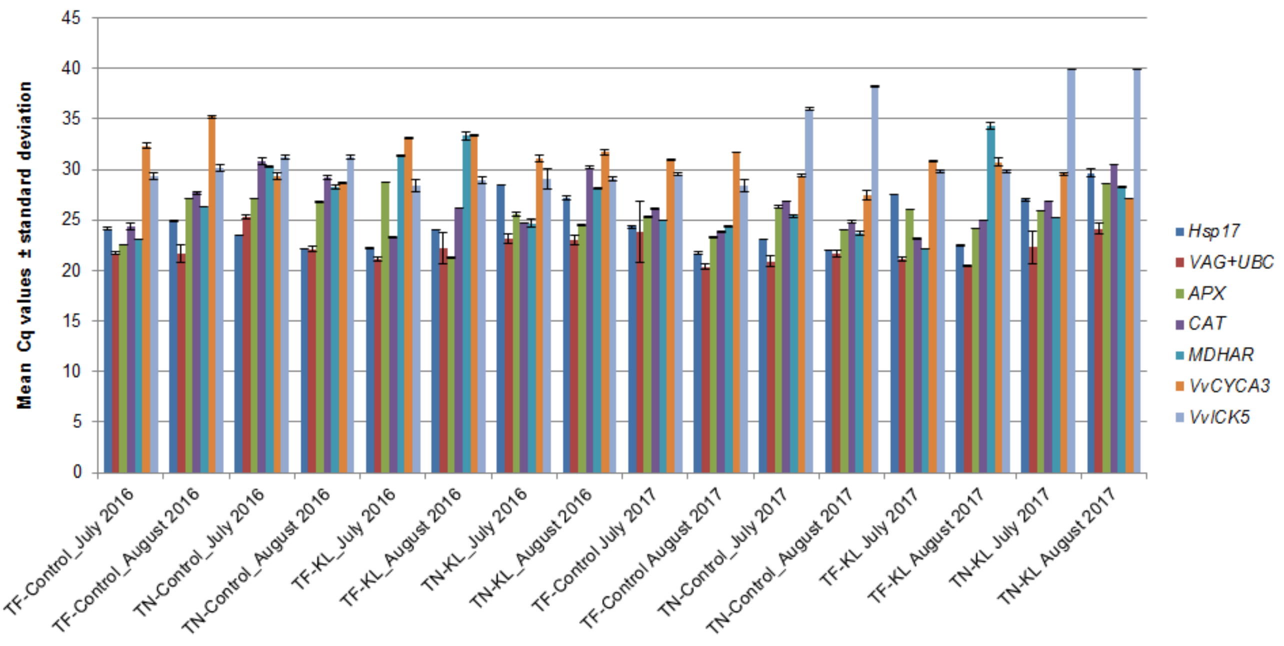

a

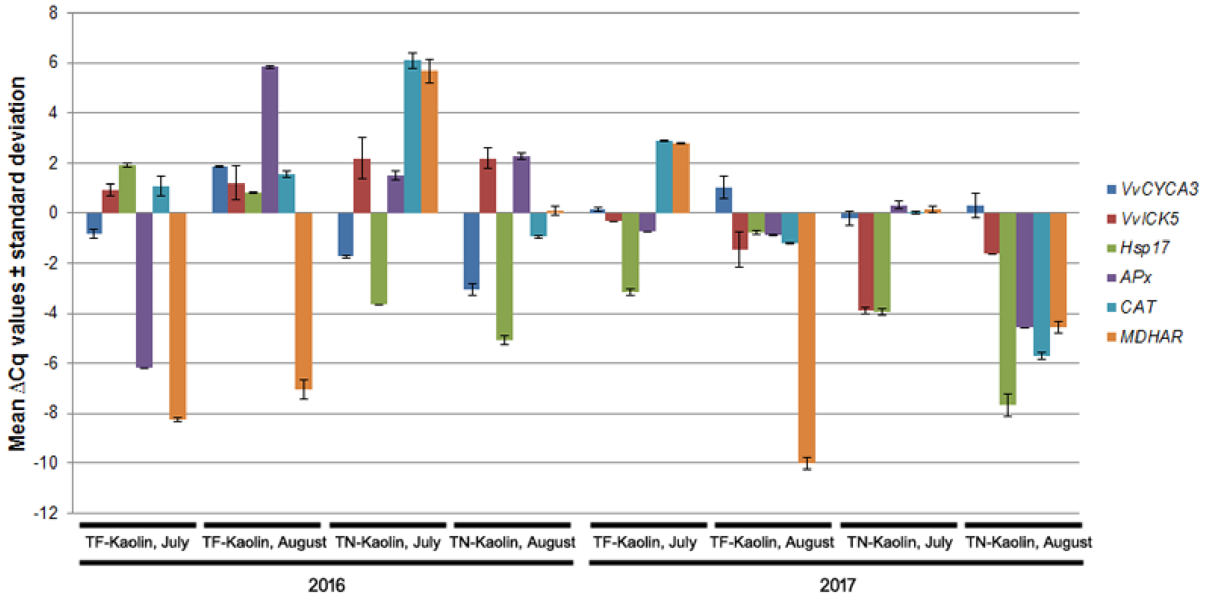

b

Figure S3. (a) Mean Cq (± standard deviation) values resulting from two biological and technical replicates (n=2) of the reference (*VAG* and *UBC*) and target (*Hsp17*, *VvCYCA3*, *VvICK5*, *APx*, *CAT* and *MDHAR*) genes; and respective (b) normalised mean ΔCq (± standard deviation) values, that were used for the calculation of the relative expression ratio of each target gene per variety × kaolin × sampling date interaction.
